# 1-Meth­oxy-11*H*-benzo[*b*]fluoren-11-one

**DOI:** 10.1107/S1600536812050076

**Published:** 2012-12-12

**Authors:** Sin-Kai Fang, Che-Wei Chang, Hsing-Yang Tsai, Ming-Hui Luo, Kew-Yu Chen

**Affiliations:** a Department of Chemical Engineering, Feng Chia University, 40724 Taichung, Taiwan

## Abstract

In the title compound, C_18_H_12_O_2_, the non-H atoms are nearly coplanar, the maximum atomic deviation being 0.113 (2) Å. π–π stacking is observed in the crystal structure, the shortest centroid–centroid distance being 3.5983 (19) Å. The mol­ecular packing is further stabilized by weak C—H⋯O hydrogen bonds, forming an infinite chain along [100] and generating a *C*(6) motif.

## Related literature

For the preparation of the title compound, see: Tang *et al.* (2011 ▶). For applications of indanone derivatives, see: Borbone *et al.* (2011 ▶); Borge *et al.* (2010 ▶); Cai & Dolbier (2005 ▶); Cui *et al.* (2009 ▶); Fu & Wang (2008 ▶); Li *et al.* (2009 ▶); Rahman *et al.* (2011 ▶); Sousa *et al.* (2011 ▶); Yu *et al.* (2011 ▶). For related structures, see: Chang & Chen (2012 ▶); Chen *et al.* (2011*a* ▶,*b* ▶).
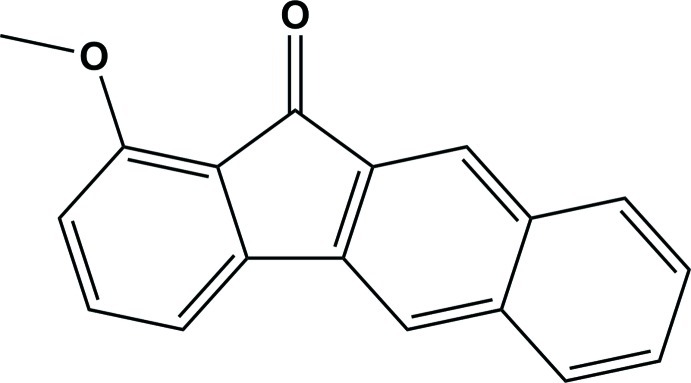


## Experimental

### 

#### Crystal data

C_18_H_12_O_2_
*M* *_r_* = 260.28
Monoclinic, 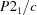

*a* = 7.7202 (3) Å
*b* = 9.2462 (4) Å
*c* = 18.0294 (8) Å
β = 99.935 (2)°
*V* = 1267.68 (9) Å^3^
*Z* = 4
Mo *K*α radiation
μ = 0.09 mm^−1^
*T* = 299 K
0.50 × 0.47 × 0.20 mm

#### Data collection

Bruker SMART CCD detector diffractometer
Absorption correction: multi-scan (*SADABS*; Bruker, 2001 ▶) *T*_min_ = 0.957, *T*_max_ = 0.983
11580 measured reflections
2559 independent reflections
1881 reflections with *I* > 2σ(*I*)
*R*_int_ = 0.042

#### Refinement

*R*[*F* ^2^ > 2σ(*F* ^2^)] = 0.083
*wR*(*F* ^2^) = 0.250
*S* = 1.11
2559 reflections
183 parameters
H-atom parameters constrained
Δρ_max_ = 0.49 e Å^−3^
Δρ_min_ = −0.42 e Å^−3^

### 

Data collection: *SMART* (Bruker, 2005 ▶); cell refinement: *SAINT* (Bruker, 2005 ▶); data reduction: *SAINT*; program(s) used to solve structure: *SHELXS97* (Sheldrick, 2008 ▶); program(s) used to refine structure: *SHELXL97* (Sheldrick, 2008 ▶); molecular graphics: *ORTEP-3 for Windows* (Farrugia, 2012 ▶); software used to prepare material for publication: *WinGX* (Farrugia, 2012 ▶).

## Supplementary Material

Crystal structure: contains datablock(s) I, global. DOI: 10.1107/S1600536812050076/xu5629sup1.cif

Structure factors: contains datablock(s) I. DOI: 10.1107/S1600536812050076/xu5629Isup2.hkl

Supplementary material file. DOI: 10.1107/S1600536812050076/xu5629Isup3.cml

Additional supplementary materials:  crystallographic information; 3D view; checkCIF report

## Figures and Tables

**Table 1:** Hydrogen-bond geometry (Å, °)

*D*—H⋯*A*	*D*—H	H⋯*A*	*D*⋯*A*	*D*—H⋯*A*
C7—H7⋯O1^i^	0.93	2.57	3.299 (5)	135
C11—H11⋯O1^ii^	0.93	2.55	3.298 (4)	138

Symmetry codes: (i) 
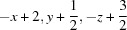
; (ii) 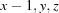.

**Fig. 1.**
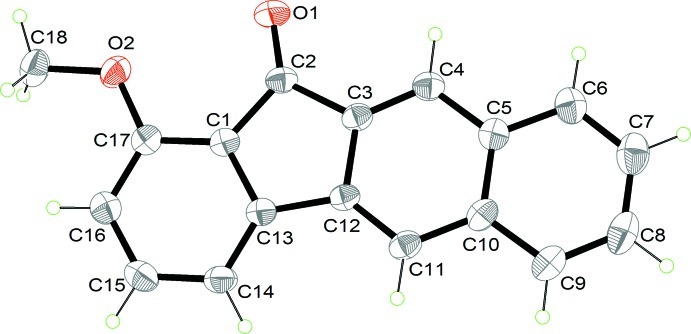
The molecular structure of the title compound, showing 50% probability displacement ellipsoids.

**Fig. 2.**
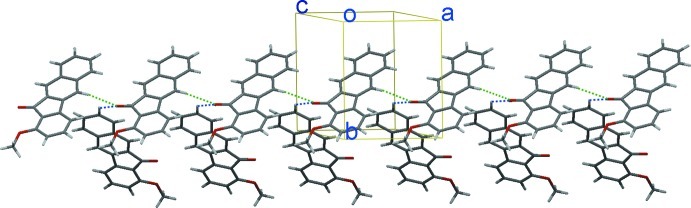
A section of the crystal packing of the title compound, viewed down the c axis. Blue and green dashed lines denote the intermolecular C7—H7···O1 and C11—H11···O1 hydrogen bonds, respectively.

## supplementary crystallographic information

### Comment

Indanone and its derivatives are some of the most widely used organic compounds (Rahman *et al.*, 2011). They are used as pigments and dyes (Cui *et al.*, 2009; Li *et al.*, 2009), intermediates in organic synthesis (Borbone *et al.*, 2011; Borge *et al.*, 2010; Fu & Wang, 2008; Yu *et al.*, 2011) and exhibit a wide variety of biological activities (Sousa *et al.*, 2011). In addition, 1-indanones were important precursors in the regiospecific synthesis of 2-fluoro-1-naphthols (Cai *et al.*, 2005).

The molecular structure of the title compound comprises a 7-methoxy-1-indanone unit having a naphthalene ring fused on one side (Fig. 1). The 1-indaneone moiety is essentially planar (r.m.s. deviation = 0.0075 Å), which is consistent with previous studies (Chang & Chen, 2012; Chen *et al.*, 2011*a*,*b*). π—π stacking is observed between the tetracyclic plane and its adjacent one, the closest centroid-centroid distance being 3.5983 (19) Å [symmetry code: 2 - *x*, -*y*, 2 - *z*]. The molecular packing (Fig. 2) is further stabilized by weak non-classical intermolecular C—H···O hydrogen bonds (Table 1).

### Experimental

The title compound was synthesized according to the literature (Tang *et al.*, 2011). Yellow parallelepiped-shaped crystals suitable for the crystallographic studies reported here were isolated over a period of six weeks by slow evaporation from a chloroform solution.

### Refinement

The C bound H atoms were positioned geometrically (C—H = 0.93–0.96 Å) and allowed to ride on their parent atoms, with *U*_iso_(H) = 1.2*U*_eq_(C).

### Crystal data

**Table:**

C_18_H_12_O_2_	*F*(000) = 544
*M**_r_* = 260.28	*D*_x_ = 1.364 Mg m^−^^3^
Monoclinic, *P*2_1_/*c*	Mo *K*α radiation, λ = 0.71073 Å
Hall symbol: -P 2ybc	Cell parameters from 6867 reflections
*a* = 7.7202 (3) Å	θ = 2.7–26.4°
*b* = 9.2462 (4) Å	µ = 0.09 mm^−^^1^
*c* = 18.0294 (8) Å	*T* = 299 K
β = 99.935 (2)°	Parallelepiped, yellow
*V* = 1267.68 (9) Å^3^	0.50 × 0.47 × 0.20 mm
*Z* = 4	

### Data collection

**Table:**

Bruker SMART CCD detector diffractometer	2559 independent reflections
Radiation source: fine-focus sealed tube	1881 reflections with *I* > 2σ(*I*)
Graphite monochromator	*R*_int_ = 0.042
φ and ω scans	θ_max_ = 26.4°, θ_min_ = 2.3°
Absorption correction: multi-scan (*SADABS*; Bruker, 2001)	*h* = −9→9
*T*_min_ = 0.957, *T*_max_ = 0.983	*k* = −11→10
11580 measured reflections	*l* = −22→21

### Refinement

**Table:**

Refinement on *F*^2^	Secondary atom site location: difference Fourier map
Least-squares matrix: full	Hydrogen site location: inferred from neighbouring sites
*R*[*F*^2^ > 2σ(*F*^2^)] = 0.083	H-atom parameters constrained
*wR*(*F*^2^) = 0.250	*w* = 1/[σ^2^(*F*_o_^2^) + (0.1046*P*)^2^ + 1.682*P*] where *P* = (*F*_o_^2^ + 2*F*_c_^2^)/3
*S* = 1.11	(Δ/σ)_max_ < 0.001
2559 reflections	Δρ_max_ = 0.49 e Å^−^^3^
183 parameters	Δρ_min_ = −0.42 e Å^−^^3^
0 restraints	Extinction correction: *SHELXL97* (Sheldrick, 2008), Fc^*^=kFc[1+0.001xFc^2^λ^3^/sin(2θ)]^-1/4^
Primary atom site location: structure-invariant direct methods	Extinction coefficient: 0.041 (7)

### Special details

**Table:**

Geometry. All e.s.d.'s (except the e.s.d. in the dihedral angle between two l.s. planes) are estimated using the full covariance matrix. The cell e.s.d.'s are taken into account individually in the estimation of e.s.d.'s in distances, angles and torsion angles; correlations between e.s.d.'s in cell parameters are only used when they are defined by crystal symmetry. An approximate (isotropic) treatment of cell e.s.d.'s is used for estimating e.s.d.'s involving l.s. planes.
Refinement. Refinement of *F*^2^ against ALL reflections. The weighted *R*-factor *wR* and goodness of fit *S* are based on *F*^2^, conventional *R*-factors *R* are based on *F*, with *F* set to zero for negative *F*^2^. The threshold expression of *F*^2^ > σ(*F*^2^) is used only for calculating *R*-factors(gt) *etc*. and is not relevant to the choice of reflections for refinement. *R*-factors based on *F*^2^ are statistically about twice as large as those based on *F*, and *R*- factors based on ALL data will be even larger.

### Fractional atomic coordinates and isotropic or equivalent isotropic displacement parameters (Å^2^)

**Table:**

	*x*	*y*	*z*	*U*_iso_*/*U*_eq_	
O1	1.2838 (3)	0.3051 (3)	0.92283 (15)	0.0675 (8)	
O2	1.3420 (3)	0.0972 (3)	1.04942 (14)	0.0571 (7)	
C1	1.0841 (4)	0.2264 (3)	1.00540 (17)	0.0428 (7)	
C2	1.1419 (4)	0.3058 (4)	0.94252 (18)	0.0464 (8)	
C3	0.9836 (4)	0.3911 (3)	0.90635 (17)	0.0439 (7)	
C4	0.9640 (4)	0.4820 (4)	0.84656 (18)	0.0490 (8)	
H4	1.0571	0.4978	0.8209	0.059*	
C5	0.8006 (4)	0.5531 (3)	0.82323 (17)	0.0461 (8)	
C6	0.7726 (5)	0.6517 (4)	0.7623 (2)	0.0579 (9)	
H6	0.8634	0.6712	0.7360	0.069*	
C7	0.6147 (6)	0.7183 (4)	0.7419 (2)	0.0683 (11)	
H7	0.5990	0.7835	0.7020	0.082*	
C8	0.4773 (6)	0.6901 (4)	0.7796 (2)	0.0694 (11)	
H8	0.3696	0.7359	0.7646	0.083*	
C9	0.4978 (5)	0.5959 (4)	0.8385 (2)	0.0578 (9)	
H9	0.4038	0.5780	0.8633	0.069*	
C10	0.6602 (4)	0.5249 (3)	0.86266 (19)	0.0475 (8)	
C11	0.6857 (4)	0.4280 (3)	0.92502 (19)	0.0473 (8)	
H11	0.5937	0.4089	0.9508	0.057*	
C12	0.8438 (4)	0.3637 (3)	0.94670 (17)	0.0401 (7)	
C13	0.9079 (4)	0.2608 (3)	1.00807 (17)	0.0420 (7)	
C14	0.8220 (4)	0.2001 (4)	1.0611 (2)	0.0522 (8)	
H14	0.7052	0.2224	1.0625	0.063*	
C15	0.9155 (5)	0.1040 (4)	1.1126 (2)	0.0589 (9)	
H15	0.8600	0.0626	1.1493	0.071*	
C16	1.0877 (5)	0.0687 (4)	1.1106 (2)	0.0544 (9)	
H16	1.1465	0.0040	1.1458	0.065*	
C17	1.1749 (4)	0.1289 (4)	1.05646 (18)	0.0471 (8)	
C18	1.4337 (5)	−0.0107 (4)	1.0985 (2)	0.0627 (10)	
H18A	1.4545	0.0248	1.1493	0.094*	
H18B	1.5440	−0.0321	1.0832	0.094*	
H18C	1.3638	−0.0971	1.0958	0.094*	

### Atomic displacement parameters (Å^2^)

**Table:**

	*U*^11^	*U*^22^	*U*^33^	*U*^12^	*U*^13^	*U*^23^
O1	0.0431 (14)	0.087 (2)	0.0777 (18)	0.0056 (12)	0.0246 (12)	0.0121 (15)
O2	0.0438 (13)	0.0598 (16)	0.0666 (16)	0.0075 (11)	0.0062 (11)	0.0054 (12)
C1	0.0410 (16)	0.0411 (16)	0.0463 (16)	−0.0028 (13)	0.0079 (12)	−0.0059 (13)
C2	0.0366 (16)	0.0548 (19)	0.0490 (17)	−0.0035 (13)	0.0105 (13)	−0.0041 (14)
C3	0.0381 (16)	0.0436 (17)	0.0502 (17)	−0.0034 (12)	0.0086 (12)	−0.0051 (13)
C4	0.0439 (17)	0.053 (2)	0.0506 (18)	−0.0039 (14)	0.0105 (13)	−0.0026 (14)
C5	0.0506 (18)	0.0394 (17)	0.0468 (17)	−0.0019 (13)	0.0040 (13)	−0.0069 (13)
C6	0.069 (2)	0.051 (2)	0.0510 (19)	0.0002 (17)	0.0046 (16)	−0.0012 (15)
C7	0.085 (3)	0.054 (2)	0.060 (2)	0.012 (2)	−0.003 (2)	−0.0058 (18)
C8	0.070 (3)	0.057 (2)	0.073 (2)	0.0191 (19)	−0.009 (2)	−0.0058 (19)
C9	0.0480 (19)	0.056 (2)	0.067 (2)	0.0071 (16)	0.0021 (16)	−0.0088 (17)
C10	0.0422 (17)	0.0401 (17)	0.0579 (18)	0.0022 (13)	0.0023 (13)	−0.0100 (14)
C11	0.0393 (16)	0.0428 (18)	0.0606 (19)	−0.0022 (13)	0.0105 (13)	−0.0071 (14)
C12	0.0355 (15)	0.0377 (16)	0.0478 (16)	−0.0042 (12)	0.0095 (12)	−0.0070 (12)
C13	0.0426 (16)	0.0360 (16)	0.0486 (16)	−0.0038 (12)	0.0113 (12)	−0.0073 (12)
C14	0.0449 (18)	0.055 (2)	0.0601 (19)	−0.0025 (14)	0.0189 (14)	−0.0008 (16)
C15	0.065 (2)	0.058 (2)	0.058 (2)	−0.0087 (17)	0.0212 (17)	0.0047 (17)
C16	0.058 (2)	0.052 (2)	0.0530 (19)	−0.0010 (16)	0.0095 (15)	0.0011 (15)
C17	0.0447 (17)	0.0449 (18)	0.0511 (17)	−0.0018 (13)	0.0064 (13)	−0.0043 (14)
C18	0.053 (2)	0.056 (2)	0.074 (2)	0.0063 (16)	−0.0035 (17)	0.0071 (18)

### Geometric parameters (Å, º)

**Table:**

O1—C2	1.208 (4)	C8—H8	0.9300
O2—C17	1.350 (4)	C9—C10	1.416 (5)
O2—C18	1.436 (4)	C9—H9	0.9300
C1—C17	1.389 (5)	C10—C11	1.425 (5)
C1—C13	1.406 (4)	C11—C12	1.354 (4)
C1—C2	1.483 (4)	C11—H11	0.9300
C2—C3	1.505 (4)	C12—C13	1.478 (4)
C3—C4	1.355 (5)	C13—C14	1.374 (4)
C3—C12	1.424 (4)	C14—C15	1.394 (5)
C4—C5	1.421 (5)	C14—H14	0.9300
C4—H4	0.9300	C15—C16	1.375 (5)
C5—C6	1.415 (5)	C15—H15	0.9300
C5—C10	1.419 (5)	C16—C17	1.394 (5)
C6—C7	1.359 (5)	C16—H16	0.9300
C6—H6	0.9300	C18—H18A	0.9600
C7—C8	1.379 (6)	C18—H18B	0.9600
C7—H7	0.9300	C18—H18C	0.9600
C8—C9	1.363 (6)		
			
C17—O2—C18	118.0 (3)	C9—C10—C11	121.9 (3)
C17—C1—C13	120.3 (3)	C5—C10—C11	119.8 (3)
C17—C1—C2	130.1 (3)	C12—C11—C10	119.9 (3)
C13—C1—C2	109.5 (3)	C12—C11—H11	120.0
O1—C2—C1	129.0 (3)	C10—C11—H11	120.0
O1—C2—C3	125.8 (3)	C11—C12—C3	120.0 (3)
C1—C2—C3	105.2 (3)	C11—C12—C13	131.9 (3)
C4—C3—C12	121.8 (3)	C3—C12—C13	108.2 (3)
C4—C3—C2	129.8 (3)	C14—C13—C1	121.2 (3)
C12—C3—C2	108.4 (3)	C14—C13—C12	130.1 (3)
C3—C4—C5	119.6 (3)	C1—C13—C12	108.7 (3)
C3—C4—H4	120.2	C13—C14—C15	117.9 (3)
C5—C4—H4	120.2	C13—C14—H14	121.1
C6—C5—C10	118.7 (3)	C15—C14—H14	121.1
C6—C5—C4	122.4 (3)	C16—C15—C14	121.8 (3)
C10—C5—C4	118.9 (3)	C16—C15—H15	119.1
C7—C6—C5	120.8 (4)	C14—C15—H15	119.1
C7—C6—H6	119.6	C15—C16—C17	120.6 (3)
C5—C6—H6	119.6	C15—C16—H16	119.7
C6—C7—C8	120.8 (4)	C17—C16—H16	119.7
C6—C7—H7	119.6	O2—C17—C1	117.4 (3)
C8—C7—H7	119.6	O2—C17—C16	124.3 (3)
C9—C8—C7	120.6 (4)	C1—C17—C16	118.3 (3)
C9—C8—H8	119.7	O2—C18—H18A	109.5
C7—C8—H8	119.7	O2—C18—H18B	109.5
C8—C9—C10	120.9 (4)	H18A—C18—H18B	109.5
C8—C9—H9	119.5	O2—C18—H18C	109.5
C10—C9—H9	119.5	H18A—C18—H18C	109.5
C9—C10—C5	118.2 (3)	H18B—C18—H18C	109.5
			
C17—C1—C2—O1	−1.7 (6)	C10—C11—C12—C13	179.8 (3)
C13—C1—C2—O1	179.4 (3)	C4—C3—C12—C11	0.8 (5)
C17—C1—C2—C3	178.3 (3)	C2—C3—C12—C11	−179.9 (3)
C13—C1—C2—C3	−0.6 (3)	C4—C3—C12—C13	−179.9 (3)
O1—C2—C3—C4	0.0 (6)	C2—C3—C12—C13	−0.6 (3)
C1—C2—C3—C4	180.0 (3)	C17—C1—C13—C14	0.4 (5)
O1—C2—C3—C12	−179.3 (3)	C2—C1—C13—C14	179.4 (3)
C1—C2—C3—C12	0.7 (3)	C17—C1—C13—C12	−178.7 (3)
C12—C3—C4—C5	0.3 (5)	C2—C1—C13—C12	0.2 (3)
C2—C3—C4—C5	−178.9 (3)	C11—C12—C13—C14	0.4 (6)
C3—C4—C5—C6	178.6 (3)	C3—C12—C13—C14	−178.8 (3)
C3—C4—C5—C10	−1.1 (5)	C11—C12—C13—C1	179.4 (3)
C10—C5—C6—C7	0.0 (5)	C3—C12—C13—C1	0.2 (3)
C4—C5—C6—C7	−179.6 (3)	C1—C13—C14—C15	0.5 (5)
C5—C6—C7—C8	−0.7 (6)	C12—C13—C14—C15	179.4 (3)
C6—C7—C8—C9	0.6 (6)	C13—C14—C15—C16	−0.7 (5)
C7—C8—C9—C10	0.1 (6)	C14—C15—C16—C17	0.1 (6)
C8—C9—C10—C5	−0.7 (5)	C18—O2—C17—C1	−175.9 (3)
C8—C9—C10—C11	178.8 (3)	C18—O2—C17—C16	3.1 (5)
C6—C5—C10—C9	0.7 (4)	C13—C1—C17—O2	178.0 (3)
C4—C5—C10—C9	−179.7 (3)	C2—C1—C17—O2	−0.7 (5)
C6—C5—C10—C11	−178.9 (3)	C13—C1—C17—C16	−1.0 (5)
C4—C5—C10—C11	0.7 (4)	C2—C1—C17—C16	−179.7 (3)
C9—C10—C11—C12	−179.2 (3)	C15—C16—C17—O2	−178.2 (3)
C5—C10—C11—C12	0.3 (5)	C15—C16—C17—C1	0.8 (5)
C10—C11—C12—C3	−1.1 (5)		

### Hydrogen-bond geometry (Å, º)

**Table:**

*D*—H···*A*	*D*—H	H···*A*	*D*···*A*	*D*—H···*A*
C7—H7···O1^i^	0.93	2.57	3.299 (5)	135
C11—H11···O1^ii^	0.93	2.55	3.298 (4)	138

Symmetry codes: (i) −*x*+2, *y*+1/2, −*z*+3/2; (ii) *x*−1, *y*, *z*.
